# Association of NT-proBNP and Multiple Biomarkers with Severity of Angiographic Coronary Artery Disease in Diabetic and Pre-Diabetic Chinese Patients

**DOI:** 10.1371/journal.pone.0022563

**Published:** 2011-08-16

**Authors:** Zhou Fang, Linuo Zhou, Yuanyuan Bao, Wei Ding, Haiming Shi, Xinping Luo, Renming Hu

**Affiliations:** 1 Department of Endocrinology and Metabolism, Huashan Hospital of Fudan University, Shanghai, China; 2 Institute of Endocrinology and Diabeteology, Shanghai, China; 3 Department of Cardiology, Huashan Hospital at Fudan University, Shanghai, China; 4 State Key Laboratory of Genetic Engineering, Shanghai, China; University of Tor Vergata, Italy

## Abstract

**Background:**

Little is known about the plasma levels of N-terminal pro-brain natriuretic peptide (NT-proBNP), and the relationship between the severity of coronary heart disease (CHD) with NT-proBNP and multiple biomarkers in diabetic and pre-diabetic patients, compared to individuals with normal glucose levels.

**Methods:**

Four hundred and fifteen consecutive Chinese patients of both sexes were assigned to three groups on the basis of the new hemoglobin (Hb) A1c (HbA1c) cut-off points for diagnosis of diabetes and pre-diabetes. The three groups were divided into tertiles according to NT-proBNP, hs-CRP, cystatin C, and troponin T levels. Gensini scores were compared among the three groups and biomarker tertiles. Receiver operating characteristic (ROC) curves were used to obtain the angiographic CHD cut-off points for each biomarker. Stepwise multivariate linear correlation analysis was applied to examine the association between the severity of CHD and biomarker levels.

**Results:**

Gensini scores increased with increasing biomarker tertile levels and HbA1c. Gensini scores were significantly different in the middle and upper NT-proBNP tertiles of the diabetic, pre-diabetic and control groups. NT-proBNP had the highest positive and negative predictive values and area under the curve for CHD. Only NT-proBNP was identified as an independent variable for Gensini score.

**Conclusions:**

Plasma NT-proBNP may be an important biomarker to evaluate the severity of CHD and screen for CHD in diabetic or pre-diabetic patients.

## Introduction

Coronary heart disease (CHD) is the main cause of morbidity and mortality in developed countries, and the prevalence is increasing in developing countries. In China, nearly two thirds of chronic heart failure is the result of CHD [1]. Several studies have reported biomarker clusters which are associated with CHD. The assessment of these biomarkers, alone or in combination, may improve the long-term prediction of mortality or first major cardiovascular event compared to conventional risk markers [2], [3].

N-terminal pro-brain natriuretic peptide (NT-proBNP) was identified as a novel and important CHD biomarker, and has prognostic value in patients with stable CHD [4]. A report from the BELSTRESS study suggested that NT-proBNP levels were a strong predictor of coronary events in over 10,000 working men after 2.66 years, even after adjustment for conventional risk factors [5]. However, recently two studies reported that NT-proBNP was not more predictive of long-term CVD and CVD outcomes in individuals without pre-existing CHD than conventional risk factors [6], [7]. This suggests that NT-proBNP may be more suitable for screening individuals with preexisting CHD.

Inflammation plays a role in the pathogenesis of CHD, and cystatin C and high-sensitive C-reactive protein (hs-CRP) may act as markers of inflammation [8], [9]. cystatin C is a newer biomarker associated with ischemia independent of kidney function, and may contribute to atherosclerotic plaque vulnerability [8]. Hs-CRP is one of the most studied non-traditional biomarkers for the evaluation of CHD risk [10], [11].

Diabetes is an established risk factor for CHD [12]; however, it remains unknown whether plasma BNP levels and other biomarkers are increased in diabetic and pre-diabetic patients with coronary artery stenosis. In this study, we aimed to determine whether plasma levels of NT-proBNP and other biomarkers altered with changes in the severity of CHD, and whether these markers are appropriate for rapidly identifying symptomatic or asymptomatic CHD in diabetic or pre-diabetic Chinese patients.

## Methods

### Ethics statement

The present study was approved by the Ethics Committee of the Huashan Hospital, Shanghai, China. Verbal informed consent was obtained from all patients. The study was a cross-sectional study performed in inpatients.

### Patients

Four hundred and fifteen consecutive symptomatic or nonsymptomatic Chinese patients scheduled for coronary angiography for suspected myocardial ischemia were recruited between February 2008 and March 2009 at the Huashan Hospital of Fudan University, China. Patients with valvular disease, dilated or hypertrophic cardiomyopathy, hyperthyroidism and hypothyroidism were excluded from the study to eliminate potential confounding factors which may influence heart function and plasma biomarkers. The patients were divided into diabetic (including previous history of diabetes), pre-diabetic, and control groups according to the new HbA1c cut-off points for diagnosis of diabetes or pre-diabetes [13]. A fasting venous blood sample was obtained for measurement of fasting glucose and HbA1c. Patients with HbA1c levels ≥5.7% to 6.4% were considered pre-diabetic; patients with HbA1c levels ≥6.5% were diagnosed as diabetic, even without previous history of diabetes. In each group, the subjects were subdivided into tertiles based on plasma levels of NT-proBNP, troponin T, hs-CRP and cystatin C. Subjects were interviewed for medical history (e.g., hypertension and diabetes) and smoking habits. Body weight and height were measured to determine the body mass index (BMI; BMI, kg/m^2^ = weight (kg)/[height (m)]^2^). Blood pressure was measured by standard methods.

### Laboratory assays

Venous blood was collected after overnight fasting before coronary artery angiography. Fasting plasma glucose (FPG) was quantified by the glucose oxidase procedure; HbA1c was measured by ion-exchange high-performance liquid chromatography (HPLC; Bio-Rad, Hercules, CA, USA). The homeostasis model assessment insulin resistance estimate (HOMA-IR) was calculated as serum glucose (mg/dL)×plasma insulin (µU/mL)/22.5, serum total cholesterol (TC), high-density lipoprotein (HDL) cholesterol, triglyceride (TG) levels, apolipoprotein A1 (apo-AI) and B (apo-B), lipoprotein (a) (LP(a)), creatinine (Cr), and uric acid (UA) were measured by an enzymatic method with a chemical analyzer (Hitachi 7600-020, Tokyo, Japan). Low-density lipoprotein (LDL) cholesterol was calculated using the Friedewald formula, and creatinine clearance rate (Ccr) was calculated using the Cockcroft-Gault formula. An enzyme coupled method was used to measure urine creatinine concentration and to calculate the urinary albumin/urinary creatinine ratio (Alb/Cr). The electrochemiluminescence-based immunoanalytical system, Elecsys 2010 (Roche Diagnostics Ltd., Mannheim, Germany) was used to determine plasma levels of NT-proBNP, plasma insulin, C peptide, and urinary albumin. Immunoturbidimetry was used to determine serum cystatin C, hs-CRP, and troponin I. The day-to-day and inter-assay coefficients of variation at the central laboratory in our hospital for all analyses were between 1% and 3%.

### Echocardiography

Two-dimensional and Doppler echocardiography scans were performed using the HP77020A echocardiograph (Hewlett Packard Company, USA). The aortic dimension (AOD), left atrial dimension (LAD), interventricular septal thickness (IVST), posterior wall thickness (PWT), left ventricular end-diastolic diameter (LVEDd) and ejection fraction (EF) percentage were assessed. The left ventricular mass index (LVMI) was calculated using the Devereux equation. LVM (g) was calculated using 1.04×[(IVSTd+LVPWTd+LVEDd)^3^−(LVEDd)^3^]−13.6. Body surface area (BSA, m^2^) was calculated using 0.0061×height (m)+0.0128×body weight (kg)−0.1529; LVMI (g /m^2^) = LVM/BSA.

### Angiographic assessments

A catheterizing cardiologist and qualified and experienced angiographer performed the angiographic assessments, while blinded to the patient biomarker levels. Coronary angiography was performed the Judkins technique. Angiographic coronary artery disease (CAD) was defined as a stenotic lesion of at least 50% in one or more major coronary arteries or the main coronary artery. The severity of angiographic CHD was quantified precisely using the Gensini score [14].

### Statistical analysis

Data were expressed as mean ± SD for continuous variables, or as percentages for categorical variables. Statistical analysis was performed using SPSS for Windows (Version 13.0). NT-proBNP and Alb/Cr values were logarithmically transformed as they were not normally distributed. Age, sex, and smoking were adjusted by covariance analysis. Repeated measures one-way ANOVA was used to determine the significance of trends within groups. Numerical data was compared using the chi-square test. Receiver operating characteristic (ROC) curves and Youden indexes were used to obtain the biomarker cut-off points for predicting the prevalence of angiographic CHD. The respective areas under the curve (AUC), sensitivity, specificity, positive predictive value, and negative predictive value were compared among biomarkers, and in total. Stepwise multivariate linear correlation analysis was applied to examine the association between the severity of CHD, test factors and each biomarker.

## Results

### Demographic and metabolic characteristics of subjects

Of the 415 patients, age, hypertension, BMI, SBP, HOMA-IR, Loge alb/Cr, LVMI and Gensini score were significantly different between groups and rose progressively from control group<pre-diabetes group<diabetes group (Table 1). HDL cholesterol level decreased gradually in the three groups. The percentage of patients with previous CHD, confirmed by cardiac angiography or known myocardial infarction within the past 6 years, and the type of anti-hypertensive treatment or anti-lipid treatment did not differ significantly among the control, pre-diabetic and diabetic groups.

**Table 1:** Characteristics of the control, pre-diabetic and diabetic study groups.

Groups	Control (n = 137)	Pre-diabetes(n = 149)	Diabetes(n = 129)	*P*-value
Age (Mean year ± SD)	65.01±11.23	67.60±10.89	69.09±10.34	0.008
Males % (n)	59.9 (82/137)	65.1 (97/149)	60.5 (78/129)	>0.05
Current smokers % (n)	25.6 (35/137)	26.8 (40/149)	25.6 (33/129)	>0.05
Hypertension % (n)	64.9 (89/137)	68.5 (102/149)	81.4 (105/129)	<0.01
BMI (Mean kg/m^2^ ± SD)	24.24±3.50	24.54±3.55	25.47±3.55	0.013
SBP (Mean mmHg ± SD)	130.96±17.18	130.75±18.75	137.62±19.73	0.003
DBP (Mean mmHg ± SD)	77.70±10.78	76.55±10.30	78.06±1.40	0.509
FPG (Mean mmol/L± SD)	5.03±0.68	5.45±0.81	7.35±2.54	0.000
HbAlc (Mean % ± SD)	5.37±0.23	5.954±0.20	7.28±1.27	0.000
HOMA-IR (Mean % ± SD)	2.51±1.63	2.87±1.97	4.74±4.19	0.000
Fasting insulin (Mean µg/L ± SD)	10.67±6.88	11.57±7.88	13.27±8.24	0.050
Fasting C peptide (Mean ng/L ± SD)	2.03±1.096	2.14±1.06	2.30±1.64	0.329
TC (Mean mmol/L± SD)	4.37±1.017	4.55±1.00	4.55±1.20	0.287
TG (Mean mmol/L± SD)	1.56±0.86	1.74±1.44	1.92±1.17	0.061
HDL (Mean mmol/L± SD)	1.12±0.29	1.11±0.24	1.04±0.26	0.029
LDL (Mean mmol/L± SD)	2.65±0.93	2.75±0.86	2.75±1.01	0.609
APO-AI (Mean ng/L ± SD)	1.02±0.16	1.02±0.15	0.99±0.16	0.275
APO-B(Mean ng/L ± SD)	0.74±0.23	0.80±0.23	0.80±0.26	0.135
LP(a) (Mean ng/L ± SD)	160.17±136.35	200.39±163.61	175.74±158.16	0.079
Log_e_ alb/Cr (Mean ± SD)	2.10±0.77	2.41±1.24	2.95±1.54	0.000
BUN (Mean mmol/L± SD)	5.80±1.86	6.10±1.67	6.15±2.04	0.251
Cr (Mean mmol/L± SD)	77.96±18.18	78.56±17.71	80.29±26.36	0.646
Ccr (Mean % ± SD)	77.48±26.57	76.06±26.06	76.25±30.27	0.903
UA (Mean mmol/L ± SD)	0.34±0.10	0.36±0.10	0.33±0.08	0.075
Heart rate (Mean bmp ± SD)	72.09±10.06	71.12±10.11	73.33±10.75	0.253
EF (Mean % ± SD)	64.70±9.51	63.04±10.38	62.15±11.29	0.130
LVMI(Mean kg/m^2^ ± SD)	119.12±35.10	125.74±49.80	134.46±36.14	0.011
Gensini score (Mean ± SD)	22.67±36.10	25.94±32.96	33.63±36.57	0.043
Previous CHD % (N)	11.7 (16/137)	14.1 (21/149)	14.7 (19/129)	>0.05
Anti-hypertension treatment % (N)	29.9 (41/137)	30.9 (46/149)	40.3 (52/129)	>0.05
Anti-lipids treatment % (N)	40.2 (55/137)	49.0 (73/149)	55.8 (72/129)	>0.05

### Comparison of NT-proBNP, troponin T, hs-CRP, and cystatin C with the severity of CHD in the control, pre-diabetic, and diabetic patients

**Figure 1:**
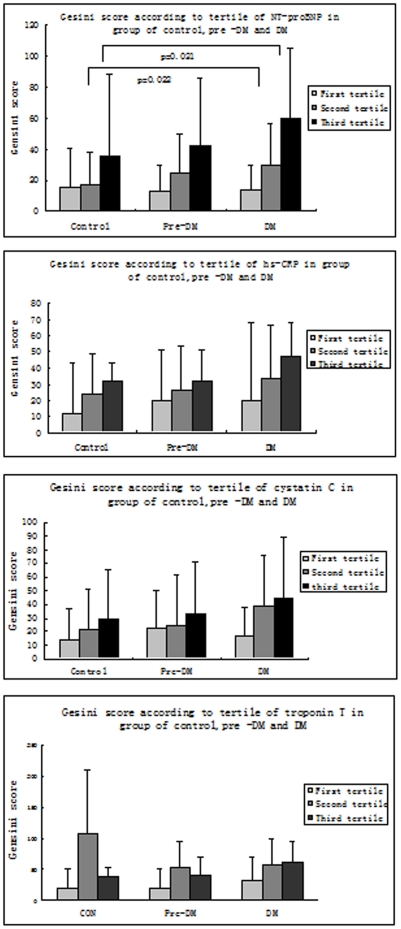
Gensini scores were compared among the three groups and biomarker tertiles. Scores were calculated according to (A) NT-proBNP, (B) hs-CRP (C) cystatin C and (D) troponin T tertiles in the control, pre-DM and DM groups. Pre-DM: pre-diabetes, DM: diabetes mellitus.

There was a progressive and graded relationship between Gensini score and NT-proBNP, hs-CRP and cystatin C tertiles from the control to the pre-diabetic and diabetic group (Fig. 1 A–C). The differences in Gensini score were significant among the tertiles of all groups; The differences in troponin T among patient groups were significant; however, the differences in the tertile subgroups were not significant. The Gensini score among the diabetic, pre-diabetic and control groups in the second and third tertiles was significantly related to NT-proBNP levels, and these differences remained significant after covariance analysis adjustment for age, sex, and smoking habits.

### Diagnostic power of NT-proBNP, troponin T, hs-CRP, and cystatin C for angiographic CHD

**Figure 2:**
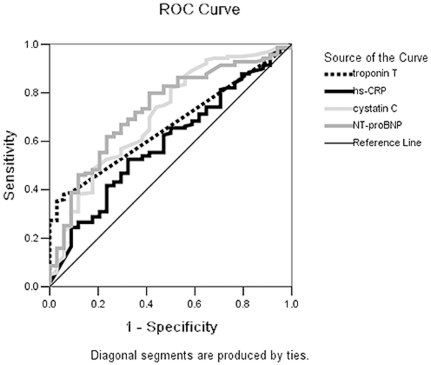
Receiver operating characteristic curves to predict angiographic coronary heart disease in hospitalized high-risk Chinese patients. ROC curves show NT-proBNP, troponin T, hs-CRP and cystatin C for the prediction of angiographic coronary heart disease (CHD) in hospitalized high-risk Chinese patients.

The respective cut-off points for diagnosis of CHD were estimated according to the ROC curves and the Youden indexes for NT-proBNP, troponin T, hs-CRP, and cystatin C (Fig. 2). Using these cut-off points, NT-proBNP showed higher sensitivity, specificity, positive and negative predictive values with a greater area under the ROC curve than other biomarkers, except for the specificity and positive predictive value of troponin T. By combining the four biomarkers we obtained a slightly greater area under the ROC curve and higher positive predictive value for CHD, using the ROC curves and Youden index, (Table 2).

**Table 2:** Youden index cut-off points, sensitivity, specificity, positive predictive value, negative predictive value, and area under the curves for biomarkers and combined number of biomarker abnormalities.

Biomarker	Cut-off point	Sensitivity(%)	Specificity(%)	*PPV(%)	**NPV(%)	Area underROC curve
NT-proBNP	78.9 pg/mL	80.0	44.4	79.4	44.8	0.69
Cystatin C	0.78 mg/L	81.8	52.8	76.8	34.4	0.67
Troponin T	0.015 µg/L	38.0	91.7	95.3	34.9	0.65
Hs-CRP	1.98 mg/L	52.5	67	78.9	33.3	0.58
Combined number of biomarker abnormalities	>1.5 items#	65.0	62.7	88.0	42.6	0.75

*Positive predictive value;

**Negative predictive value,

#Higher than cut-off points.

### Correlation of NT-proBNP and multiple biomarkers with severity of angiographic CAD in diabetic, pre-diabetic and control groups

The correlation of NT-proBNP and multiple biomarkers with angiographic CAD in the diabetic and pre-diabetic groups was obtained through linear stepwise regression analysis (See Table S1). In the diabetic, pre-diabetic and control groups, as well as the total study population, NT-proBNP manifested as significant variable which contributed to the Gensini score. All other biomarkers, including troponin T, hs-CRP, and cystatin C, were not identified as independent variables. Other independent variables contributing to Gensini score were age, BMI, HOMA-IR, TC, HDL, apo-AI, LP(a), Cr, Ccr (%), EF, and LVMI, in different combinations.

## Discussion

In this study, in which the new HbA1c criteria were applied for the diagnosis of pre-diabetes and diabetes, we observed a progressive and graded relationship between NT-proBNP, troponin T, hs-CRP and cystatin C tertile levels and the severity of angiographic CHD in high-risk patients. The severity of angiographic CHD was increased in diabetic and pre-diabetic patients in the middle and upper NT-proBNP tertiles. To our knowledge, only few similar studies have been conducted, and this is the first report in a Chinese population.

There is evidence to suggest that NT-pro-BNP levels may reflect increased left ventricular wall stress, in the absence of cardiac ischemia, thus, NT-pro-BNP can reflect heart function and heart hypertrophy, as well being closely related to CHD [15]. Furthermore, NT-proBNP concentrations increased proportionally to the size of myocardial infarct estimated using scintigraphy, despite the absence of heart failure in asymptomatic myocardial infarct patients [16]. Elevated BNP may be associated with a greater severity and degree of myocardial ischemia, and may partly explain the association between elevated BNP levels and adverse outcomes [17]. Several studies have revealed that NT-proBNP levels reflect the extent of ischemia in patients with stable CAD accompanied by episodes of ischemia [18] and patients with exercise-induced myocardial ischemia [19]. Our findings are in agreement with the results of several previous studies [18], [19] where NT-proBNP plasma levels were closely related to the severity of angiographic CHD. Furthermore, in our study, the mean levels of NT-proBNP in the middle and top tertiles were greater than the cut-off point (78.9 pg/mL) for diagnosis of angiographic CHD (Table 2), indicating the severity of CHD increased gradually in tertile groups, as well as from the control to the pre-diabetic and diabetic groups.

In recent years, the ability of troponin I, cystatin C, and hs-CRP to predict risk of death or cardiovascular events has been evaluated in addition to NT-proBNP [3]. Hs-CRP is one of the most studied non-traditional biomarkers for the CHD risk. One study rigorously evaluated the predictive utility of other novel risk markers such as CRP, excluding NT-proBNP, and indicated that they offer minimal improvement over the Framingham Risk Score [20]; however, another suggested that hs-CRP, combined with NT-proBNP, troponin I, and cystatin C, is associated with CHD when the influence of age is excluded, and in niche populations [21]. It has been recognized that hs-CRP and cystatin C are markers of inflammation and that cystatin C is associated with ischemia. Our study found that hs-CRP and cystatin C are related to Gensini score; however, the AUC, positive predictive value, and negative predictive value of hs-CRP and cystatin C were lower than NT-proBNP. Furthermore, unlike NT-proBNP, the biomarkers hs-CRP, cystatin C, and troponin T were not identified as independent variables in stepwise linear models to assess the relationship with existing CHD. This finding suggests hs-CRP, cystatin C, and troponin T have a weak or indirect relationship with the severity of coronary arterial stenosis in the control, pre-diabetes, or diabetes groups. Troponin I, similar to troponin T in structure and clinical significance, indicates myocardial necrosis and is an established “gold standard” for diagnosis of myocardial infarction, explaining the high specificity and positive predictive value of Troponin T for diagnosis of CHD. We hypothesis that Troponin T may not increase proportionally with Gensini score as the severity of necrosis is not equal to the severity of coronary arterial stenosis.

Our results also revealed moderate efficacy (and no improvement) in sensitivity and specificity using a single biomarker, such as NT-proBNP, to screen or diagnose angiographic CHD. Although coronary angiography is considered the gold standard for evaluation of the degree of coronary stenosis, some researchers have indicated angiography is not as sensitive as computed tomography angiography (CTA) or intravascular ultrasound. As these techniques allow visualization of atherosclerosis, they are particularly valuable for accurate early identification of CAD [22]. We assume that the sensitivity and even specificity of NT-proBNP may correlate better with CTA and intravascular ultrasound results than coronary artery angiography findings.

It is well known that glucose level is directly linked to CVD risk. The Reykjavik Study and a meta-analysis suggested a slightly stronger association between CHD risk and HbA1c levels than fasting or post-load glucose levels [23]. A prospective observational study (UKPDS-35) revealed that every 1% reduction in baseline HbA1c levels decreases the incidence of myocardial infarction by 5% [24]. In 2010, the American Diabetes Association (ADA) formally recommended new HbA1c criteria for the diagnosis of diabetes and pre-diabetes [15], and these criteria were used in our study. Additionally, as symptomatic hospitalized patients experience physiological and psychological stress, which may increase blood glucose levels, use of HbA1c levels categorize hyperglycemia in our study was reasonable. Progressive narrowing of the coronary arteries was observed in all 3 groups, from individuals with HbA1c levels <5.7%, to pre-diabetes at 5.7–6.4%, and diabetics with HbA1c ≥6.5%. This observation is in agreement with the findings of numerous studies, including a previous study by our group [25], where the increase in CHD was investigated pre-diabetic and diabetic individuals using glucose levels. As a marker of CHD, NT-proBNP showed the same trend which increased with regard to the severity or prevalence of CHD.

In summary, we demonstrated that plasma NT-proBNP levels are closely related to the severity of CHD in a Chinese population of diabetic and pre-diabetic patients. The relationship between NT-proBNP and CHD was stronger in diabetic and pre-diabetic patients than normoglycemic controls. NT-proBNP is a stronger predictor of CHD than other biomarkers such as troponin T, hs-CRP, and cystatin C, which have been suggested to be predictors of CHD risk. As a diagnostic tool, plasma NT-proBNP levels may not be sensitive and specific enough; therefore, further studies are needed to determine the potential of NT-proBNP as a biomarker of CHD.

## Supporting Information

Table S1 **Multivariate linear correlation of NT-proBNP, troponin T, hs-CRP and cystatin C plasma concentrations with Gensini score CHD severity.**
(DOC)
