# Supplementary material for: Association of NT-proBNP and Multiple Biomarkers with Severity of Angiographic Coronary Artery Disease in Diabetic and Pre-Diabetic Chinese Patients
Source: PLoS One. 2011 Aug 16;6(8):e22563. doi: 10.1371/journal.pone.0022563 (PMC3156698; doi:10.1371/journal.pone.0022563)
Supplement: Table S1 — Multivariate linear correlation of NT-proBNP, troponin T, hs-CRP and cystatin C plasma concentrations with Gensini score CHD severity. (DOC) [file pone.0022563.s001.doc]

**Table S1 Multivariate linear correlation of NT-proBNP, troponin T, hs-CRP and cystatin C plasma concentrations with Gensini score CHD severity**

| Model | Biomarkers (plus other independent variables*) | Variable* | Unstandardized coefficients  Beta | Standardized coefficients  B | *P-* value. |
| --- | --- | --- | --- | --- | --- |
| DM | Log_e_ NT-proBNP | Log_e_ NT-proBNP | 10.082 | 0.377 | 0.001 |
|  |  | LP(a) (ng/L) | 0.062 | 0.302 | 0.007 |
|  |  | BMI (Kg/m^2^) | -3.253 | -0.311 | 0.006 |
|  |  | TC (mmol/L) | -9.056 | -0.287 | 0.013 |
|  | Troponin T (ug/L) | LP(a) (ng/L) | 0.064 | 0.316 | 0.022 |
|  |  | Ccr (%) | -0.358 | -0.321 | 0.019 |
|  |  | EF (%) | -0.930 | -0.287 | 0.036 |
|  | Hs-CRP (mg/L) | Cr (mmol/L) | 0.349 | 0.281 | 0.020 |
|  |  | LP(a) (ng/L) | 0.057 | 0.279 | 0.021 |
|  | Cystatin C (mg/L) | HOMA-IR | 2.024 | 0.308 | 0.024 |
|  |  | Cr (mmol/L) | 0.310 | 0.303 | 0.026 |
| Pre-diabetes | Log_e_ NT-proBNP | Log_e_ NT-proBNP | 12.402 | 0.513 | 0.000 |
|  |  | Loge ALB/cr | -9.356 | -0.283 | 0.024 |
|  |  | HDL (mmol/L) | -48.779 | -0.258 | 0.029 |
|  | Troponin T (ug/L) | HDL (mmol/L) | 77.427 | -0.365 | 0.020 |
|  |  | Cr (mmol/L) | .704 | 0.362 | 0.022 |
|  | Hs-CRP (mg/L) | APO-A (ng/l) | -99.000 | -0.299 | 0.024 |
|  | Cystatin C (mg/L) | Cr (mmol/L) | .573 | 0.291 | 0.040 |
| Control | Log_e_ NT-proBNP | EF (%) | -4.790 | -0.737 | 0.000 |
|  |  | LVMI (Kg/m2) | 0.572 | 0.390 | 0.013 |
|  | Troponin T (ug/L) | Age (year) | 4.987 | 0.635 | 0.036 |
|  | Hs-CRP (mg/L) | EF (%) | -4.790 | -0.737 | 0.000 |
|  |  | LVMI (Kg/m2) | 0.572 | 0.390 | 0.013 |
|  | Cystatin C (mg/L) | Ccr (%) | -0.542 | -0.466 | 0.019 |
| Total | Log_e_ NT-proBNP | Log_e_ NT-proBNP | 8.105 | 0.288 | 0.000 |
|  |  | LP(a) (ng/L) | 0.056 | 0.202 | 0.006 |
|  |  | Cr (mmol/L) | 0.318 | 0.173 | 0.021 |
|  |  | EF (%) | -0.613 | -0.156 | 0.045 |
|  | Troponin T (ug/L) | LP(a) (ng/L) | 0.094 | 0.305 | 0.001 |
|  |  | EF (%) | -1.200 | -0.297 | 0.002 |
|  |  | Ccr | -0.396 | -0.247 | 0.009 |
|  | Hs-CRP (mg/L) | Cr (mmol/L) | 0.355 | 0.193 | 0.012 |
|  |  | EF (%) | -1.017 | -0.258 | 0.001 |
|  |  | LP(a) (ng/L) | 0.057 | 0.205 | 0.006 |
|  | Cystatin C (mg/L) | Cr (mmol/L) | 0.441 | 0.302 | 0.001 |
|  |  | HOMA-IR | 1.842 | 0.176 | 0.040 |

*Other independent variables: Gensini score; variables: Age, BMI, SBP, DBP, FPG, HbAlc (%), HOMA-IR, fasting insulin, fasting C peptide, TC, TG, HDL, LDL, Apo-AI, Apo-B, LP (a), Cr, log_e_ alb/Cr, BUN, Cr, Ccr (%), UA, EF (%), LVMI.
